# Beyond Efficacy: Preclinical Validation and Translational Readiness of Recent Multimodal Photodynamic Nanoplatforms for Glioblastoma

**DOI:** 10.3390/ijms27219878

**Published:** 2026-11-04

**Authors:** Paweł Woźnicki, David Aebisher

**Affiliations:** 1 Doctoral School, Faculty of Medicine, Collegium Medicum, University of Rzeszów, 35-310 Rzeszów, Poland; pawelw@dokt.ur.edu.pl; 2 Department of Photomedicine and Physical Chemistry, Faculty of Medicine, Collegium Medicum, University of Rzeszów, 35-310 Rzeszów, Poland

**Keywords:** glioblastoma, photodynamic therapy, nanoplatforms, nanomedicine, preclinical models, biodistribution, pharmacokinetics, biosafety, translational readiness, multimodal therapy

## Abstract

Multimodal photodynamic therapy (PDT)-based nanoplatforms are increasingly being developed for glioblastoma (GBM) to improve photosensitizer delivery, overcome tumor hypoxia, and combine PDT with complementary therapeutic mechanisms. However, promising antitumor activity does not necessarily translate into adequate preclinical validation or clinical readiness. This review critically evaluates recent studies published between January 2025 and August 2026, identified through PubMed, Scopus and Web of Science. Of 3617 records screened, 35 studies met the broader eligibility criteria for nanoparticle-mediated PDT in GBM, including 19 studies incorporating in vivo evaluation. Particular attention was given to the biological relevance of experimental models, survival assessment, biodistribution, pharmacokinetics, clearance, systemic and neurological safety, and platform-specific translational limitations. Orthotopic models were used in 16 of 19 in vivo studies, whereas immunocompetent and immunodeficient hosts were represented almost equally. Nevertheless, clinically challenging settings such as post-resection disease, acquired temozolomide resistance and recurrence after standard therapy were rarely reproduced. Quantitative pharmacokinetic and elimination data were limited, and fluorescence or elemental measurements frequently reflected individual platform components rather than intact nanostructures. Safety assessments were predominantly short-term and often focused on body weight, routine blood parameters and organ histology. Overall, the current evidence demonstrates substantial therapeutic innovation but also reveals a persistent gap between antitumor efficacy and translational validation. Future studies should prioritize clinically representative models, quantitative absorption, distribution, metabolism and excretion (ADME) characterization, long-term and neurological safety assessment, and comparisons with current standard-of-care treatment.

## 1. Introduction

Glioblastoma (GBM) remains one of the most aggressive tumors of the central nervous system, and the prognosis for patients remains poor despite the use of maximally safe resection, radiotherapy and temozolomide (TMZ) [1]. The effectiveness of treatment is limited by, amongst other things, the infiltrative nature of the tumor growth, the tumor’s significant heterogeneity, the development of treatment resistance and the high rate of recurrence [2]. An additional challenge is posed by the blood–brain barrier (BBB) and the blood–tumor barrier (BBTB), whose variable permeability can significantly limit and spatially vary the delivery of drugs to GBM [3,4].

Photodynamic therapy (PDT) is an attractive approach due to the possibility of controlling the cytotoxic effect both spatially and temporally through the activation of a photosensitizer by light [5]. However, its use in GBM is hampered by the limited delivery of photosensitizers to the tumor, the poor penetration of light into brain tissue, and hypoxia in the tumor microenvironment [5,6]. In response to these limitations, nanoplatforms are being developed to improve the transport, accumulation and activity of photosensitizers, and modern systems are increasingly combining PDT with additional mechanisms, such as photothermal therapy, chemotherapy, ferroptosis, modulation of metabolism and hypoxia, immunotherapy, gene editing and targeted protein degradation [6,7].

However, the increasing functional complexity of nanoplatforms does not necessarily mean they are ready for clinical translation. Strong in vitro cytotoxicity or tumor growth inhibition in a short-term animal experiment does not yet answer questions regarding the adequacy of the model used, pharmacokinetics, biodistribution, elimination, the integrity of the nanoplatform in vivo, neurological and systemic safety, or the consequences of repeated dosing [8,9]. This is of particular significance in GBM, where the efficacy demonstrated in simple experimental models may not reflect the conditions of residual disease following resection, treatment resistance or recurrence [10].

This review evaluates the preclinical validation and translational readiness of recent multimodal PDT-based nanoplatforms for glioblastoma. Literature published between January 2025 and August 2026 was searched in PubMed, Scopus and Web of Science using the Boolean query “PDT AND nanoparticles”. Original experimental studies investigating GBM and employing a nanostructured PDT-based therapeutic platform were included, whereas reviews, non-GBM studies and studies without PDT or a nanostructured therapeutic system were excluded. Of 3617 records identified, 35 studies met the eligibility criteria, of which 19 included in vivo evaluation and constituted the primary focus of the translational analysis. These studies were critically assessed with regard to the relevance of the experimental models, the strength of the in vivo evidence, biodistribution, pharmacokinetics and clearance, systemic and neurological safety, and platform-specific translational limitations. The aim was to identify not only promising directions for further development, but also key evidence gaps that should be addressed before these systems can progress toward clinical translation.

## 2. Preclinical Evidence Landscape and Relevance of In Vivo Models

The translational significance of new therapeutic strategies being developed for the treatment of GBM depends not only on their anti-tumor efficacy, but also on the extent to which the experimental models used reflect the biological, anatomical and therapeutic characteristics of the disease observed in patients [11,12]. Of the 35 original studies included in the review, 19 (54.3 per cent) involved in vivo assessments, whilst 16 (45.7 per cent) were limited to in vitro studies. This means that almost half of the studies analyzed have not yet provided data from animal models. This has significant translational implications, as results concerning cytotoxicity, cellular uptake or the mechanism of action obtained in vitro do not allow for the assessment of many parameters relevant to the efficacy, safety and translational potential of GBM therapy, such as pharmacokinetics and systemic distribution [13], transport across the blood–brain barrier and the blood–tumor barrier [14,15], accumulation within the tumor [16], interactions with the host’s immune system [17] or systemic toxicity [16]. For this reason, the subsequent analysis focuses on 19 studies in which the experimental assessment was extended to in vivo models.

This emphasis on in vivo validation should not be interpreted as diminishing the value of simpler experimental models. Rather, preclinical GBM models should be viewed as complementary levels within a progressive evidence hierarchy, each addressing different questions. Conventional two-dimensional cell cultures remain useful for the rapid and relatively inexpensive screening of cytotoxicity, cellular uptake, photodynamic activity, mechanism of action and optimization of formulation parameters. More complex three-dimensional systems, including tumor spheroids and organoid-based models, can provide additional information on cell–cell interactions, spatial heterogeneity, treatment penetration, and responses within a tissue-like architecture while retaining greater experimental control than animal models. In vivo models subsequently add systemic pharmacokinetics, biodistribution, host–tumor interactions, immune responses and treatment-related toxicity, which cannot be reproduced comprehensively in vitro [11,12]. Accordingly, translational progression should not be understood as the replacement of simple models by complex ones, but as sequential validation in increasingly informative systems, with the complexity of the model matched to the question being addressed.

A significant advantage of the available in vivo data was the relatively frequent use of orthotopic GBM models. At least one orthotopic model was used in 16 of the 19 studies (84.2 per cent), whilst three studies (15.8 per cent) assessed therapeutic efficacy exclusively in subcutaneous tumors: Zhang et al. [18], Li et al. [19] and Besbinar et al. [20]. Jiang et al. used both subcutaneous and orthotopic U87-MG models [21], whereas He et al. used an additional subcutaneous model for imaging studies, while therapeutic efficacy was assessed in an orthotopic GL261 model [22]. The predominance of orthotopic models is a key factor in enhancing the biological and translational relevance of preclinical data on GBM [23]. The intracranial implantation of cancer cells makes it possible to at least partially recreate the specific conditions of the brain’s microenvironment [24], the physical limitations of the intracranial space [25], interactions with nerve cells and resident immune cells [24,26] and—in the case of systemically administered platforms—the need to reach the tumor by crossing the blood–brain barrier and/or the blood–tumor barrier [15,23]. However, the presence of an intracranial tumor does not imply that systemically administered material necessarily crosses an intact BBB. Stereotactic implantation itself may transiently alter local vascular and tissue permeability, whereas subsequent tumor growth can progressively remodel the neurovascular interface and generate a spatially heterogeneous blood–tumor barrier (BBTB) [27]. Importantly, barrier integrity may be substantially reduced within parts of the tumor core while remaining relatively preserved in the infiltrative tumor margin and the surrounding brain. Consequently, increased accumulation of a nanoplatform in an orthotopic tumor should not, by itself, be interpreted as evidence of efficient transport across an intact BBB; the permeability state of the tumor and surrounding brain should be considered when evaluating brain-delivery claims [14,15]. Subcutaneous models remain useful for initial proof-of-concept assessments, particularly given their ability to facilitate the easy and quantitative monitoring of tumor growth inhibition; however, they do not replicate a number of key conditions characteristic of GBM developing within the CNS [28]. For this reason, strong anti-tumor activity demonstrated solely in a subcutaneous model should not be regarded as equivalent to demonstrating efficacy in an intracranial GBM model [29].

The in vivo studies analyzed were almost evenly divided in terms of the host’s immune status. In 9 of the 19 studies (47.4 per cent), immunocompetent mice, mainly C57BL/6 or C57BL/6J, were used in syngeneic models employing GL261 or CT2A cells [30]. This group also included two models based on the Sleeping Beauty system: one generated in C57BL/6J mice [31], and a second study using SB mGSCs implanted intracranially into C57 mice [32]. In ten studies (52.6%), however, immunocompromised animals were used, including BALB/c nude mice, NOD/SCID mice or other strains described as ‘nude’ [33,34]. This group was dominated by xenografts of human GBM cells, including, amongst others, U87/U87MG, U251 and LN229, whilst one study used C6 rat cells implanted into nude mice [18]. The biological relevance of the implanted cell line is an additional determinant of model validity. Long-established serum-cultured GBM cell lines, including U87MG, U251 and LN229, remain useful because of their experimental reproducibility, ease of culture and reliable tumor formation; however, they do not fully reproduce the molecular and phenotypic heterogeneity of patient GBM [11,12]. In particular, the widely used ATCC U87MG line has been shown to differ genetically from the original U87 cells established in Uppsala, and is currently regarded as a human glioblastoma cell line of uncertain origin [35]. Their use is therefore appropriate for reproducible proof-of-concept and comparative studies, but the translational strength of efficacy data should be interpreted in the context of these biological limitations. Both categories of models address different research questions. Human cell xenografts enable the assessment of the platform’s efficacy against human GBM cells in vivo [36]. However, the use of hosts with compromised immunity significantly limits the ability to analyze the full spectrum of interactions between the tumor and the immune system [36,37]. In contrast, syngeneic models retain the host’s immunocompetence [30] and are particularly useful in the evaluation of platforms designed to induce immunogenic cell death [38], the modulation of immune cells present in the tumor microenvironment [39], or for use in combination with immunotherapy [40]. This distinction is particularly important for photoimmunotherapy strategies and platforms—including certain biomimetic systems—whose mechanism of action involves the modulation of immune cells or the remodeling of the tumor’s immune microenvironment [41,42]. Beyond host immune status, the biological origin of the tumor model also affects translational relevance. Patient-derived xenografts (PDXs) can preserve important histological, molecular and treatment-response characteristics of the donor tumor more faithfully than long-established cell-line xenografts, making them valuable for modeling interpatient heterogeneity and evaluating therapies in a more clinically representative human GBM background [43,44]. Their major limitation is the requirement for immunodeficient hosts, which restricts the assessment of tumor–immune interactions and is particularly relevant when evaluating immunomodulatory PDT-based strategies [43]. Genetically engineered and transposon-based models, including Sleeping Beauty-derived GBM, offer a complementary advantage by enabling tumor development in an immunocompetent host and by introducing defined oncogenic alterations in situ [43,45]. However, their genetically defined nature and murine origin may not fully reproduce the genomic, phenotypic and interpatient heterogeneity of human GBM. Consequently, PDX and genetically engineered models should be viewed as complementary rather than interchangeable approaches, with model selection guided by the biological and translational question being addressed.

**Figure 1:**
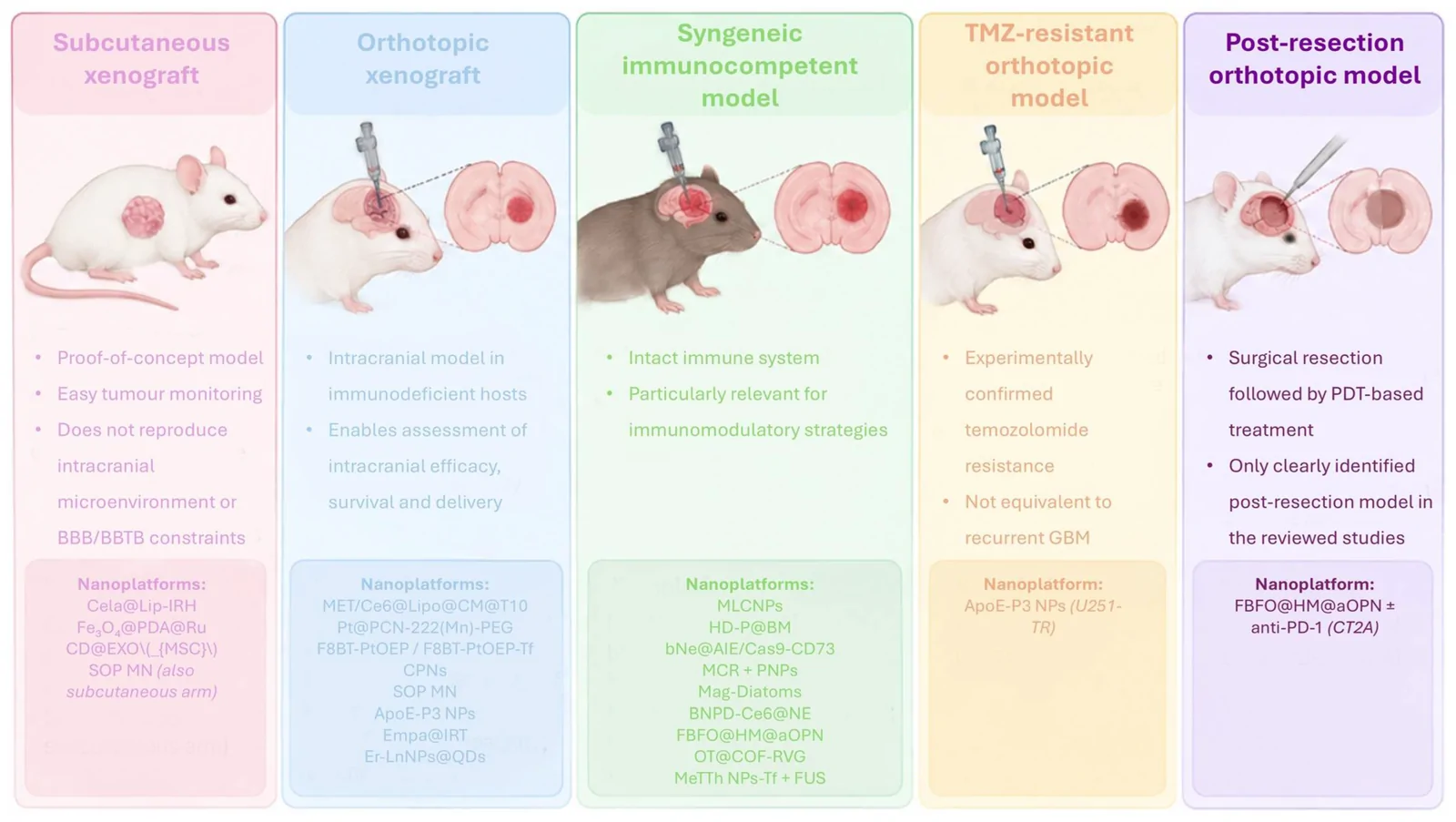
In vivo glioblastoma models used for the preclinical evaluation of multimodal PDT-based nanoplatforms.

Despite the widespread use of orthotopic models, clinically, more complex forms of GBM have been modeled much less frequently. Su et al. utilized an orthotopic model established from TMZ-resistant U251-TR cells, providing data under conditions of experimentally confirmed resistance to this drug [46]. This represents a significant extension compared with models based solely on previously untreated cells. It should be emphasized, however, that the resistance already present in the implanted cell population cannot be equated with recurrent GBM developing following prior multimodal treatment [47,48]. This is because such a model does not replicate the molecular and phenotypic evolution of the tumor associated with previous treatment [48], changes in the immune microenvironment observed upon relapse [49] or post-operative residual disease arising following tumor resection [47]. Similarly, only Zhao et al. provided a definitive assessment of treatment in an orthotopic model following tumor resection. In that study, CT2A tumors were surgically removed prior to the administration of nanoparticle-based therapy, including in combination with an anti-PD-1 antibody [50]. Such an experimental design more closely reflects the clinical situation in which, following the surgical removal of the main tumor mass, infiltrating tumor cells may remain in the surrounding brain tissue [51]. At the same time, other models should not be classified as post-operative without unequivocal confirmation that a resection has been performed. Particular caution is required when interpreting the study by Xu et al. Although the authors justified the development of the therapy by the need to treat small residual lesions remaining after the surgical resection of GBM, they evaluated the therapy experimentally in a model of a small orthotopic tumor without a prior resection having been performed [52]. This model should therefore not be equated with the model of post-operative residual disease [53]. Even greater caution is required when interpreting the study by Jiang et al., in which the microneedle platform was administered locally within the tumor. This route of administration may suggest a potential post-operative application; however, the orthotopic model used did not describe surgical resection of the tumor or the deliberate induction of residual disease prior to treatment [21]. This model should therefore not be classified as a post-operative model. Regardless of the biological validity of the model used, the size of the experimental groups was also a significant factor influencing the strength of the available evidence. The treatment groups were generally small, most often comprising around three to six animals, and the group size was not always clearly reported for each analysis carried out. Such small groups may provide preliminary data in exploratory or proof-of-concept studies; however—particularly in the absence of a formal justification for the sample size—they limit the precision of estimates of effect size and biological variability, and increase uncertainty regarding the reproducibility of the results obtained [54,55]. The in vivo models used in the reviewed studies and the corresponding PDT-based nanoplatforms are summarized in Figure 1. Considerable heterogeneity was also observed in terms of the duration of observation and the endpoints used. Survival was assessed in 11 of the 19 in vivo studies (57.9 per cent), with all studies reporting this parameter using orthotopic models. In the remaining studies, efficacy was assessed primarily on the basis of longitudinal imaging, tumor volume or mass, histopathological examinations, molecular markers, or a combination of these parameters. However, the methodology for conducting and reporting survival observations was not standardized across the individual studies. For example, Caverzan et al. monitored the animals until a humane endpoint was reached, carrying out daily assessments of neurological and general condition [56], whilst Li et al. reported survival up to day 40 [57], and Su et al. up to around day 45 in the treatment group [46]. In several studies that did not include a survival analysis, however, the therapeutic trial was terminated after approximately two to four weeks. This heterogeneity makes it difficult to directly compare the durability of the therapeutic response across different platforms [47]. In particular, a short-term halt or delay in tumor growth does not equate to achieving long-term control of the disease [58].

Longitudinal imaging should be interpreted according to the biological source of the detected signal. In models using luciferase-expressing tumor cells, bioluminescence imaging was used primarily as an indirect measure of intracranial tumor burden and treatment response, because the signal originates from viable tumor cells expressing luciferase [59,60]. By contrast, the fluorescence imaging of labeled nanoplatforms or their components was used primarily to assess the spatial and temporal distribution of the administered material. Such fluorescence therefore reflects the distribution of the fluorescent label or labeled component, rather than tumor burden itself, and its interpretation requires separate validation [59,61]. These approaches are thus complementary but address different questions: bioluminescence primarily monitors tumor progression, whereas nanoplatform-associated fluorescence primarily monitors the distribution of labelled material. Bioluminescence-based tumor response should therefore be interpreted together with survival and clinical or neurological endpoints, while fluorescence-based distribution should be considered together with quantitative biodistribution and pharmacokinetic data. Additional endpoints, including, amongst others, apoptosis, proliferation, hypoxia, the immunological composition of the tumor microenvironment and its remodeling, supported a mechanistic interpretation of the results in some of the studies; however, the set of parameters analyzed and the timing of their assessment varied significantly between individual studies.

A significant limitation in assessing the translational potential of the analyzed nanoplatforms is the scope of the therapeutic comparisons used. The studies were primarily based on controls that were either untreated or received PBS, radiotherapy alone, and variants of the platforms differing in composition or light activation. In none of the 19 studies analyzed was a direct comparison identified with a regimen comprising resection followed by radiotherapy with concurrent and adjuvant TMZ. Nor were separate arms for radiotherapy or free TMZ monotherapy identified in vivo [62]. Even in Zhao et al.’s post-resection model, the comparisons involved the nanoplatform, the anti-PD-1 antibody and a combination of the two, with no reference to chemoradiotherapy [50]. Similarly, Yan et al. used a T@COF-RVG variant containing TMZ; however, this comparison concerned modifications made to the composition of the nanosystem, rather than its efficacy compared with conventionally administered free TMZ [63]. In total, two studies examined separate anti-PD-1 arms and its combination with a nanoplatform [50,57]. The results obtained therefore provide evidence of the anti-tumor activity of the platforms under investigation, but do not allow for a determination of their efficacy compared with the current standard of care, nor of the additional therapeutic benefit and toxicity resulting from the incorporation of the nanoplatform into that treatment. This limits the scope of translational conclusions, whilst not undermining the value of these studies as a preliminary assessment of the anticancer activity of new platforms.

In summary, 54.3% of the analyzed studies included in vivo assessments, with a clear predominance of orthotopic models (84.2%) and frequent use of immunocompetent hosts. This points to the growing importance of GBM models that are more biologically and anatomically relevant. At the same time, key elements of the clinical course of the disease, such as resistance associated with prior treatment, post-resection status, residual disease and recurrence, were still very rarely replicated. Further limitations include heterogeneous and often short follow-up periods, the insufficient analysis of survival, and a lack of direct comparisons with standard treatment. Further preclinical development should therefore combine efficient early-stage screening in experimentally tractable models with sequential validation in increasingly complex systems, culminating in in vivo models that reproduce the anatomical, immunological and therapeutic context relevant to the intended clinical application.

## 3. Bioavailability, Pharmacokinetics and Elimination

The characterization of biodistribution, pharmacokinetics and elimination is one of the key elements of the preclinical evaluation of nanoplatforms intended for the treatment of GBM [64]. In the case of nervous system therapy, it is particularly important not only to assess the delivery of the nanoplatform to the tumor site, but also to determine its circulation time, distribution in healthy brain tissue and peripheral organs, and the pathways and kinetics of its elimination from the body [65,66]. The interpretation of these parameters should take the route of administration into account, as this affects the distribution and exposure of the nanoplatform; in the case of GBM, systemic administration additionally requires the brain barriers to be crossed, whilst local delivery can bypass them and limit systemic exposure [67,68]. In the studies analyzed, intravenous administration was by far the most common method. In three studies, however, the platform was delivered locally, either directly to the brain or within the tumor: via an intracranial catheter in the study by Wu et al. [31], by intracranial injection in the case of Mag-Diatoms, as reported by Li et al. [32], and using locally implanted microneedles, as described by Jiang et al. [21]. In the study by Yan et al., intravenous administration via the caudal vein was clearly described in the safety experiments, whilst the route of administration of the nanoplatform in the main therapeutic cohort was not explicitly specified [63]. This uncertainty materially limits the translational interpretation of the therapeutic data, because the route of administration determines whether the observed efficacy also provides evidence for systemic delivery, including exposure to the circulation and the requirement to overcome BBB/BBTB constraints. Accordingly, the therapeutic results demonstrate the antitumor activity of the OT@COF-RVG system under the reported experimental conditions, but they should not be used to draw firm conclusions regarding the efficiency of systemic brain delivery or the pharmacokinetic behavior of the platform. This distinction is important because local delivery allows some of the limitations associated with systemic circulation and transport across the blood–brain barrier to be circumvented [69]; however, it does not allow for a direct assessment of the pharmacokinetics and biodistribution characteristic of systemic administration [65].

The most commonly used method for assessing distribution following intravenous administration was whole-animal fluorescence imaging, supplemented in some studies by ex vivo analysis of the brain and major organs. The time intervals used varied considerably. He et al. monitored the signal for up to 24 h [22], Jin et al. within the range of 2–24 h [70], and Zhao et al. from 1 to 24 h [50]; Zhang et al., on the other hand, monitored the fluorescence of the rhodamine B isothiocyanate (RITC) probe for up to 96 h [18]. Luo et al. carried out second near-infrared window (NIR-II) imaging up to 96 h after administration, and then assessed the signal in isolated organs [71]. This approach enables the longitudinal monitoring of relative changes in the signal and its distribution across the formulations under investigation. It should be emphasized, however, that without appropriate validation, fluorescence imaging primarily provides qualitative or semi-quantitative data, and does not constitute a fully quantitative measurement of the biodistribution of the nanomaterial [72]. Serial imaging of the brain or tumor allows the local dynamics of the signal to be tracked; however, it does not replace the serial blood or plasma measurements required to characterize systemic pharmacokinetics [73].

In selected studies, fluorescence imaging indicated the increased accumulation of labeled platform components within the brain or tumor following the use of delivery-targeting strategies. Su et al. used ApoE-P3 nanoparticles, in which the semiconducting polymer P3 formed the core, whilst the surface was modified using the DSPE–PEG–ApoE peptide with the sequence (LRKLRKRLL)_2_C [46], which mimics the apolipoprotein E region responsible for receptor binding [74]. In the case of ApoE-P3, the highest NIR-II signal in the brain was observed 24 h after administration [46]. In the study by Luo et al., the bNe@AIE/Cas9-CD73 platform, which utilizes neutrophils as carriers, produced stronger fluorescence within the tumor than AIE dots. However, ex vivo analysis after 96 h revealed a distinct signal in the liver and lungs as well, and in the AIE dots group, also in the spleen. The authors interpreted the lower fluorescence in the liver and spleen in the bNe@AIE/Cas9-CD73 group as a reduction in non-specific uptake by the reticuloendothelial system [71]. These results document differences in the distribution of the fluorescent signal, but do not directly determine the quantity of intact nanoplatforms in individual tissues. A distinction must be made between demonstrating the presence of nanoplatform components in the brain and quantitatively confirming their preferential accumulation in the tumor relative to the non-neoplastic brain parenchyma. An example of quantitative analysis at the organ level was the study by Caverzan et al., in which inductively coupled plasma mass spectrometry (ICP-MS) was used to quantify platinum derived from the PtOEP dopant, which was employed as an elemental tracer for the nanoplatform. Twenty-four hours after a single intravenous administration, Pt was detectable in brain samples from animals receiving F8BT-PtOEP-Tf CPNs, whilst in the F8BT-PtOEP CPNs group, its concentration remained below the detection limit of 1 ppb. In both groups, the concentration of Pt was higher in the liver than in the brain, although the analysis was limited to these two organs. The assay provided quantitative data on the content of the elemental tracer in the tissues; however, the entire brain was analyzed, without separate quantification of Pt in the tumor and the surrounding parenchyma [56]. The results therefore did not allow for the determination of intracerebral selectivity or the confirmation of the presence of intact nanoplatforms. Furthermore, a result below the limit of detection did not rule out a small accumulation of material in the group without transferrin functionalization. Biodistribution studies showed the presence of nanoplatform components in extra-cerebral organs as well. In the case of Pt@PCN-222(Mn)-PEG, ICP-MS analyses indicated the highest Mn content primarily in the liver and spleen, whilst measurements at subsequent time points enabled an assessment of changes in its concentration in tissues [75]. For bNe@AIE/Cas9-CD73, a distinct fluorescent signal was observed after 96 h, mainly in the liver and lungs, whilst strong fluorescence in the spleen was noted in the AIE dots control group [71]. In contrast, the analysis of F8BT-PtOEP CPNs focused exclusively on the liver and brain, revealing higher concentrations of Pt in the liver [56]. However, the presence of a signal in ex vivo imaging should not be equated with quantitative confirmation of the presence of an intact nanoplatform, nor with an assessment of its long-term retention [61]. In the case of platforms intended for multiple dosing, particularly where single-dose studies indicate persistent distribution to peripheral organs or incomplete elimination of their constituents, consideration should be given to conducting distribution studies following repeated doses and at appropriately selected subsequent time points, to enable the assessment of potential accumulation of the platform’s constituents [76].

From an analytical perspective, the biodistribution approaches used in the reviewed studies can be considered at different levels of information. At the qualitative level, in vivo fluorescence imaging primarily indicates the spatial and temporal distribution of a fluorescent label and enables relative comparisons between treatment groups. Ex vivo fluorescence imaging of excised organs, when combined with measurements such as radiant efficiency or mean fluorescence intensity, provides a semi-quantitative comparison between organs or experimental groups, but does not directly yield tissue concentrations or establish the structural integrity of the nanoplatform [61,72,77]. Quantitative elemental methods such as ICP-MS provide absolute concentrations of selected elements in tissue, and therefore offer a higher level of quantitative information; however, they do not determine whether the detected element remains incorporated within the original nanostructure or is present as ions, fragments or degradation products [78].

The assessment of nanoplatform integrity requires a different analytical strategy. Rather than relying on a single signal, orthogonal or multi-detector approaches should ideally track more than one structural component of the formulation and determine whether carrier-associated and cargo-associated signals remain co-localized or co-fractionated over time. Separation-based techniques such as size-exclusion chromatography or asymmetric-flow field-flow fractionation (AF4), coupled with complementary detectors including UV/Vis, multi-angle light scattering (MALS), dynamic light scattering (DLS) and, where appropriate, mass spectrometry, can provide information on particle size, drug loading, formulation stability and component association in complex biological matrices [79,80]. These approaches were not employed in the reviewed studies, and therefore represent an important methodological gap. Radiolabeling coupled with PET or SPECT, single-particle ICP-MS and mass spectrometry imaging may further strengthen whole-body, particle-level and spatially resolved biodistribution assessment, respectively; however, none of these methods alone necessarily demonstrates the integrity of a multicomponent nanoplatform. The most informative strategy is therefore the combination of orthogonal measurements that independently assess carrier, cargo and, where relevant, degradation products.

Future biodistribution studies could strengthen these assessments by combining complementary analytical techniques rather than relying on a single optical or elemental readout. Radiolabeling coupled with positron emission tomography (PET) or single-photon emission computed tomography (SPECT) can provide the sensitive, quantitative and longitudinal whole-body tracking of labeled nanomaterials [64,81]. However, the stability of the radiolabel and its continued association with the nanocarrier should be verified, because the redistribution of a released label may otherwise be misinterpreted as the redistribution of the nanoplatform itself [64]. Single-particle inductively coupled plasma mass spectrometry (spICP-MS) can additionally provide information on particle number concentration, particle size and elemental content for compatible inorganic nanomaterials in biological matrices, thereby complementing conventional total-element ICP-MS [82]. Spatially resolved approaches, including mass spectrometry imaging, may further provide molecular localization directly within tissue sections, and thereby complement bulk organ-level measurements [83]. Importantly, no single analytical technique necessarily establishes the structural integrity of a multicomponent nanoplatform; therefore, orthogonal approaches that independently monitor the carrier and therapeutic cargo are preferable when the in vivo fate of the complete system is the endpoint of interest [64].

Pharmacokinetic analyses provided information on the effect of the nanoplatform design on the persistence of the monitored components in the circulation. Luo et al. reported half-lives of 0.93 h for NDA-DPE, 2.23 h for AIE dots and 5.92 h for bNe@AIE/Cas9-CD73. In this comparison, the longest half-life was therefore observed for the biomimetic system utilizing bone marrow-derived neutrophils as nanoparticle carriers [71]. Su et al., however, demonstrated an increase in the elimination half-life from 0.52 h for P3 to 2.88 h for ApoE-P3; the analysis was carried out on healthy mice based on measurements of the absorbance of blood samples [46]. In the study by Hu et al., serial measurements of tetrakis(4-carboxyphenyl)porphyrin (TCPP) in blood following the administration of Pt@PCN-222(Mn)-PEG were used to calculate pharmacokinetic parameters. The half-lives of the distribution and elimination phases were 0.12 h and 2.82 h, respectively; the authors also reported the area under the concentration–time curve (AUC) and mean residence time (MRT) and separately assessed the distribution of Mn using ICP-MS [75]. These results should be interpreted as a characterization of the kinetics of the components or signals being measured, without automatically equating them with the kinetics of intact nanoplatforms [61].

Data on the excretion of one of the nanoplatform’s components were provided by Hu et al., who quantified Mn using ICP-MS in organs, as well as in urine and feces, following the intravenous administration of Pt@PCN-222(Mn)-PEG. In Figure S19, the reported values for excreted Mn were higher in urine at the 3, 6 and 12 h time points, whilst in feces they were higher at 24 and 48 h. The authors interpreted the subsequent predominance of excretion via feces as evidence suggesting the involvement of the hepatobiliary route. However, this interpretation requires a distinction to be made between the excretion of the measured element and the excretion of the intact nanoplatform. ICP-MS measurement following sample mineralization determined the total Mn content, without providing information on the integrity of the excreted nanostructure or on the chemical form of manganese prior to analysis [75]. It was therefore not possible to determine whether the detected Mn originated from intact nanoparticles, released ions, MOF fragments or other forms containing this element.

Topical delivery strategies constituted a distinct category. Microneedles were discussed by Jiang et al. [21] and Mag-Diatoms Li et al. [32], and the system administered via an intracranial catheter was described by Wu et al. [31]. The material was delivered directly to the tumor site or its immediate vicinity. This approach may reduce the dependence of effective delivery on prolonged systemic circulation and transport across the blood–brain barrier [69,84]. Consequently, conventional plasma pharmacokinetics is not a key parameter for describing the efficacy of local delivery to the tumor, although it remains important for assessing potential systemic exposure [85]. A comprehensive characterization of such systems should, however, take into account local retention and distribution away from the site of administration, the degradation or release of their components, and the potential for these components to enter the systemic circulation [85,86]. In the studies analyzed, data on these aspects were very limited or were not reported.

In summary, most of the studies analyzed showed that the monitored components of the systems under investigation could reach the tumor site, and the use of targeting modifications was often associated with an increase in the relative signal in the brain or GBM. At the same time, the quality and scope of the quantitative absorption, distribution, metabolism and excretion (ADME) data were significantly less consistent than the data on anticancer efficacy. In many studies, the assessment of biodistribution was based primarily on fluorescence imaging; quantitative pharmacokinetic parameters were reported only sporadically; the direct comparison of the tumor with healthy brain parenchyma was limited; and the comprehensive quantitative analysis of organ distribution and documented elimination were the exception rather than the rule. Accordingly, while the reviewed studies often support the preferential delivery of monitored platform components to the tumor, they provide much weaker evidence regarding the in vivo fate, pharmacokinetics and elimination of complete nanoplatforms. 

## 4. In Vivo Biosafety Assessment

In vivo safety assessment was included in most of the studies analyzed; however, its scope and depth varied significantly between the different platforms. Body weight monitoring and the final histopathological assessment of major organs were the most commonly used methods, whilst more comprehensive protocols covering hematology, serum biochemistry, neurological assessment or inflammatory response were only present in some of the studies. For this reason, the absence of overt signs of toxicity should not be interpreted as indicating an equivalent level of safety evidence for all the systems analyzed. It is particularly important to distinguish between a simple assessment of tolerability during a therapeutic trial and a separate toxicological study specifically designed to assess safety [68].

Body weight was one of the most frequently monitored parameters. In many studies, no significant weight loss was observed in treated animals, even in regimens involving several doses. However, this parameter has limited specificity in GBM models. In intracranial glioma models, weight loss may accompany tumor progression and a deterioration in the animal’s clinical condition [87], whilst in safety studies, changes in body weight are also used as an indicator of potential adverse effects of the system under investigation [68]. Maintaining a stable body weight may therefore support the conclusion that the drug is well tolerated, but does not in itself rule out other forms of toxicity, including organ-specific and immunological toxicity [68]. For a similar reason, survival curves for animals with tumors should be interpreted primarily as an endpoint for anticancer efficacy, rather than as an independent measure of safety [68]. In models of intracranial tumors, survival time is also often determined by the achievement of a defined humanitarian endpoint, based, amongst other things, on clinical deterioration and weight loss [87]. In the studies analyzed, deaths or cases of euthanasia not related to disease progression were not systematically reported as a separate endpoint.

For interpretative purposes, endpoints obtained in tumor-bearing animals should therefore be separated into three categories. Tumor growth or burden, longitudinal tumor imaging, tumor histology, molecular response markers and survival primarily provide evidence of therapeutic efficacy. Body weight, general clinical condition and routine laboratory parameters collected during therapeutic experiments provide supportive information on tolerability, but may be confounded by tumor progression or treatment response. More definitive claims regarding systemic or neurological safety require dedicated toxicology-oriented cohorts, preferably tumor-free animals or otherwise appropriately matched controls, evaluated using the intended dosing schedule and including serial hematology and clinical chemistry, the histopathology of major organs and healthy brain tissue, neurological or behavioral assessment, and post-treatment recovery follow-up. This distinction is particularly important in intracranial GBM models, where improved survival or neurological function may reflect tumor control rather than the absence of treatment-related toxicity.

Safety assessment should also encompass the phototherapeutic procedure itself, rather than the nanoplatform alone. At a minimum, the full treatment should be interpreted against both a light-only control and a nanoplatform-without-light control, allowing light-induced tissue effects, material toxicity and photoactivated toxicity to be distinguished. For locally delivered or intracranially implanted systems, an appropriate sham-procedure control is additionally important to separate treatment-related effects from tissue injury caused by insertion, implantation or local administration. Light exposure should be reported in sufficient dosimetric detail, including wavelength, irradiance or fluence rate, total fluence, exposure time and illumination geometry; when high irradiance or a photothermal component is involved, local temperature should also be monitored to exclude unintended hyperthermic damage [88,89]. In GBM, particular attention should be paid to the illuminated healthy brain and peritumoral tissue because photochemical or thermal injury in these regions may produce neurological toxicity independently of antitumor efficacy [90]. For systemically administered nanoplatforms, toxicological assessment should also be guided by biodistribution data, with the more detailed evaluation of organs showing the greatest exposure, particularly the liver and spleen when substantial accumulation is observed [66].

The monitoring of body weight and the animals’ general condition complemented the hematological and biochemical assessments, providing additional information on systemic safety [68]. However, the scope of this assessment varied between individual studies. In the study by Li et al. on HD-P@BM, a separate 10-day biocompatibility assessment was carried out, comprising measurements of ALT, AST, albumin, BUN and creatinine, as well as basic hematological parameters [57]. Su et al. assessed hematological parameters and blood biochemistry following treatment with ApoE-P3 [46]. Besbinar et al., on the other hand, measured ALT, ALP, AST, urea and creatinine on days 1, 7 and 14 following the administration of CD@EXO_msc_ or free chlorophyllin carbon dots [20]. Luo et al. presented a final assessment of the treatment groups, which also included hematological and biochemical parameters [71]. The studies cited did not report any clear abnormalities in the parameters assessed; however, differences in dosing regimens, observation periods, sampling points and the characteristics of the test animals limit the direct comparability of the tolerance profiles of the individual formulations. These results primarily support an assessment of short-term tolerability under the experimental conditions used, and do not allow conclusions to be drawn regarding safety during long-term or repeated exposure [68].

Organ histopathology formed another part of the safety assessment. Zhang et al. presented H&E stained images of the heart, liver, spleen, lungs and kidneys, without reporting any distinct pathological changes in the study groups [18]. Jiang et al. reported no significant changes in body weight and no visible histopathological damage to major organs following treatment with the SOP MN system; the histopathological assessment of the organs presented here concerned mice with a subcutaneous U87-MG tumor [21]. However, not all studies should be subject to the same scope of safety assessment. Zhang et al. presented tumor mass measurements, whilst no separately reported results on body weight monitoring were identified [18]. In the study by Wen et al., body weight was monitored; however, the histological analysis presented focused on the brain and the tumor, primarily in the context of treatment efficacy. The heart, liver, spleen, lungs and kidneys were collected in a separate biodistribution experiment for ex vivo fluorescence imaging; however, no histopathological assessment of these organs was provided to evaluate potential damage [91]. A similar distinction between the description of methods and the results presented is required in the study by Li et al. on Fe_3_O_4_@PDA@Ru. The authors stated that the H&E staining of the harvested organs had been carried out, whilst the published safety results primarily comprised body weight monitoring and measurements of the weights of major organs. The H&E images presented related to the tumor; no separate histological panel of peripheral organs was identified in the available publication or Supplementary Materials [19]. The absence of such findings should not be regarded either as evidence of organ damage or as confirmation that the tissue appearance is normal. The absence of abnormalities in routine H&E staining requires cautious interpretation. Histopathology primarily allows for the assessment of morphological changes present in the collected tissue samples at a specific point in time, and its sensitivity depends, amongst other things, on the correct collection and representativeness of the tissue samples being assessed. Histopathological assessment alone does not replace other toxicological parameters, such as hematology or clinical chemistry [92]. A normal H&E stain does not rule out functional disorders that do not give rise to detectable morphological changes; therefore, the histopathological assessment should be interpreted in conjunction with other toxicological parameters [92]. Furthermore, the short observation period does not allow for the exclusion of adverse effects that may become apparent with prolonged exposure, which justifies a separate assessment of long-term safety [68]. This limitation is of particular significance in the case of materials exhibiting long-term tissue retention, as the absence of histopathological changes in the short term does not determine their long-term safety or the biological consequences of further retention, transformation and degradation [93].

In the case of GBM treatment, a particularly important aspect of safety assessment remains the potential impact of the intervention on healthy brain parenchyma [90]. Xu et al. included the brain in the histopathological assessment of healthy mice carried out 15 days after administration of MeTTh NPs-Tf [52]. The study by Li et al. on Mag-Diatoms also presented H&E staining of the brain. However, the CD68 and TNF-α measurements carried out in this study related to the heart, liver, spleen, lungs and kidneys; therefore, they do not constitute evidence of neuroinflammation [32]. Wu et al., on the other hand, used Nissl staining to assess the brain tissue surrounding the tumor and the rotarod test to assess motor function following Empa@IRT treatment [94]. However, interpreting these results requires consideration of the role that each endpoint played in the experimental design, as histological, clinical and behavioral assessments could have served both to monitor potential neurotoxicity and to assess disease progression or the efficacy of treatment [95,96]. In the study by Caverzan et al., the daily observation of neurological symptoms was used to monitor the course of the disease and determine humanitarian endpoints, whilst brain histology focused on characterizing the tumor and the response to treatment [56]. In the study by Wu et al. (2025), the rotarod test was also used primarily as an indicator of therapeutic effect [94]. In orthotopic models, an improvement in behavior or motor function may reflect a reduction in the tumor burden and a reduction in the neurological deficits associated with its progression; therefore, it does not in itself constitute evidence of the non-neurotoxicity of the therapy used [96]. A more conclusive safety assessment should include appropriately selected control groups, enabling a distinction to be made between the consequences of tumor progression and the potential adverse effects of the nanomaterial itself, as well as the full treatment regimen [68].

The assessment of the inflammatory response provided additional information on the potential adverse effects of the interventions under investigation. Luo et al. assessed the effects of NT5E/CD73 intervention at time points of 0, 1, 3, 7, 10 and 14 days. The analysis included hematology, blood biochemistry, liver and lung histology, and the wet-to-dry weight ratio of these organs. In addition, IL-6, IL-1β and TNF-α were measured in bronchoalveolar lavage fluid (BALF) to assess the local inflammatory response in the lungs. The authors found no significant differences in the concentrations of these cytokines between the treated animals and the healthy control group at the time points analyzed [71]. However, interpreting these results is complicated by inconsistencies in the nomenclature of the formulations: the main text refers to bNe@AIE/Cas9-CD73, whilst the corresponding methods section in the Supplementary Material refers to bNe@CRISPR/Cas9-CD73, which limits the ability to unambiguously attribute the results to the complete platform containing AIEgen. Zhao et al., in turn, assessed serum levels of IL-1α, IL-1β, IL-6 and TNF-α as part of monitoring the systemic inflammatory response during treatment with FBFO@HM@aOPN [50]. These findings should be distinguished from the analysis of cytokines in BALF carried out by Luo et al., as they relate to a different biological sample and a different level of inflammatory response. The results of both studies provide data on selected inflammatory mediators, but are not equivalent to a full assessment of immunogenicity or immunotoxicity. Among other things, they do not provide conclusive evidence regarding the formation of antibodies against platform components, complement activation, hypersensitivity reactions or changes in the response following subsequent exposures [97,98]. The absence of an increase in the labelled cytokines should therefore be attributed solely to the mediators analyzed, the biological material and the time points [50,71].

In addition to the range of laboratory, histopathological and immunological tests carried out, the dosing regimen and the duration of follow-up after exposure to the nanoplatform also remain key elements of the safety assessment. The repeated administration of the nanoplatform was considered in both therapeutic trials and separate safety protocols. Su et al. used four treatment cycles every two days [46], Zhao et al. administered five doses at three-day intervals, whilst Li et al. administered Fe_3_O_4_@PDA@Ru intravenously every three days during a 14-day course of treatment [19]. Yan et al., on the other hand, carried out an assessment in healthy mice following three consecutive days of intravenous administration, covering histopathology, hematology and biochemistry [63]. These data provide limited information on the tolerability of repeated administration under the regimens used, but do not allow for the determination of the long-term effects of exposure or the relationship between successive doses, accumulation of the substance and the increase in toxicity [93,99]. Another limitation is the duration of observation. The safety assessments cited were conducted over a period of several to a dozen or so days; a further example is the study by Zhu et al., in which healthy mice were assessed after 7 or 14 days [100]. Such protocols enable the detection of short-term abnormalities in the parameters under investigation, but do not rule out delayed neurotoxicity, chronic organ damage or the consequences of the material persisting after the end of treatment [93,99,101].

The results presented therefore indicate, first and foremost, the absence of detectable abnormalities in the specified safety parameters, at the doses used and over the observation periods. Their scope does not justify a conclusion regarding the long-term safety of the nanoplatforms under investigation. For a preclinical safety claim extending beyond acute tolerability, we propose that the assessment should include a dedicated safety cohort evaluated beyond the active treatment period, with serial measurements rather than a single terminal time point. As a pragmatic minimum for research-stage GBM nanomedicines, a follow-up period of approximately four weeks after the completion of treatment should be considered, with longer observation for platforms showing prolonged tissue retention, containing slowly degradable or inorganic components, or intended for repeated administration. The minimum assessment should include body weight and clinical condition, serial hematology and serum biochemistry, the histopathology of major organs and healthy brain tissue, and neurological or behavioral evaluation appropriate to the model. Repeated-dose regimens should be assessed using the intended therapeutic schedule, with evaluation both during treatment and after a recovery period. Where biomimetic, cell-derived or immunomodulatory systems are used, inflammatory and immune responses should also be monitored. These safety data should be interpreted together with biodistribution, material retention and elimination measurements to identify delayed or cumulative toxicity (Table 1).

## 5. Platform-Specific Safety and Translational Considerations

The results presented so far indicate, above all, the short-term tolerability of the analyzed nanoplatforms in the models and experimental conditions used. However, the scope of standard tests, including body weight monitoring, hematology, biochemistry and histopathology of organs, does not fully address the safety issues arising from differences in the design, material, activation mechanism and delivery method of individual systems. Further translational assessment should therefore also take into account potential risks specific to the platform’s design [68]. The research needs discussed below do not imply that the corresponding adverse effects were observed in the experiments analyzed.

In the case of nanoplatforms containing inorganic components, key elements of the safety assessment include structural stability, the release of constituents, chemical transformations in the biological environment, and the long-term fate of both the starting material and the products of its transformation or degradation [99,102]. In the material analyzed, these issues relate, amongst other things, to systems containing iron oxides, MnO_2_, Fe_3_O_4_@BiFeO_3_ and metal–organic frameworks. However, the mere presence of an inorganic component does not in itself determine its long-term biopersistence [102]. Hu et al. demonstrated, using ICP-MS, the release of Mn from Pt@PCN-222(Mn)-PEG under conditions described by the authors as simulating the tumor microenvironment. The authors interpreted this result as evidence of TME-responsive biodegradation. However, the data presented do not unequivocally demonstrate an acceleration of this process compared with physiological conditions; it is therefore safer to regard them as evidence of Mn release under the conditions studied. Furthermore, the measurement of Mn content by ICP-MS alone does not determine its chemical form, nor does it allow the degree of degradation of the entire multi-component nanoplatform to be established [75]. Manganese-containing systems warrant particular attention because the toxicological relevance of Mn depends strongly on its chemical form, oxidation state, coordination environment and release kinetics. Although Mn is an essential trace element, the excessive accumulation of bioavailable Mn in the brain is associated with neurotoxicity, including mitochondrial dysfunction, oxidative stress and disturbances of neuronal homeostasis [103,104]. This issue is particularly relevant for GBM-directed platforms designed to accumulate within the brain. MnO_2_-based materials may undergo reductive and acid-responsive degradation in the tumor microenvironment, generating soluble Mn^2+^ species; therefore, total Mn measurements alone cannot distinguish intact MnO_2_, released Mn ions or other transformation products [105]. In contrast, manganese coordinated within porphyrin macrocycles can exhibit substantially greater kinetic stability and resistance to metal dissociation, although this property depends on the specific metalloporphyrin and should not be assumed without formulation-specific validation [106]. Accordingly, the safety assessment of Mn-containing nanoplatforms should include not only total Mn content, but also Mn speciation, release kinetics under physiological and tumor-relevant conditions, the stability of the metal–ligand complex, brain and peripheral-organ retention, elimination, and potential accumulation after repeated dosing. From a safety perspective, further characterization should therefore also cover the fate of the remaining components of the material, their potential release outside the tumor microenvironment, and the consequences of long-term and repeated exposure [102,107]. The absence of histopathological changes during the short-term follow-up period does not determine the long-term retention of the material or the potential consequences of its subsequent transformation [99].

For polymeric and lipid-based carriers, key areas for further evaluation include formulation stability in circulation, changes in particle size and surface properties, the premature release of the photosensitizer or co-formulated cargo, and the fate of degradation or breakdown products [108,109]. Stability demonstrated in a specific in vitro medium should not automatically be equated with the maintenance of structural integrity in vivo [110]. From a translational perspective, parallel tracking of the carrier and therapeutic payload would therefore be more informative for determining whether both components remain associated over time [109].

Platforms utilizing cell membranes and extracellular vesicles (EVs) require a separate characterization. In HD-P@BM, Li et al. demonstrated the preservation of the microglial membrane’s protein profile, and confirmed the presence of CD11b and VLA-4 in both the source membrane and the finished nanoplatform using Western blot analysis. The authors linked the preservation of these components to the platform’s adhesive properties and targeting capabilities [57], However, the mere detection of proteins does not allow us to determine their individual contribution to the supply to the GBM [111]. These results highlight the need to take into account the presence and functionality of key surface proteins when characterizing and controlling the quality of cell-coated systems [111]. Standardization of the cell source, membrane isolation procedures and the coating process is also important for their further development, as these stages can influence the properties of the final product [112]. In the case of EV, the characteristics of the source cells, the conditions under which they are cultured, and the method of isolating and preparing the vesicles are of particular importance, as these factors may influence their quantity, composition and biological properties [113]. In the case of EVs derived from mammalian cells, the product assessment should cover both the properties of the vesicle membranes and their endogenous biological load. This justifies the standardization of the cell source, culture conditions, isolation and purification procedures, and the characterization of the resulting EV population [113]. In the case of tumor-derived vesicles, a further consideration is the potential for biologically active components to be transferred to the recipient’s cells [114,115]. This is a potential issue that requires assessment, rather than evidence of an adverse effect of the formulations under investigation. The demonstration of tumor-targeting properties should therefore be supplemented by a characterization of the biological activity of the carrier material itself [113]. The FBFO@HM@aOPN platform combined vesicles derived from M1 macrophages with bacterial outer membrane vesicles (OMVs), whose immunostimulatory properties formed part of the intended therapeutic mechanism. The authors used the Escherichia coli W3110 ΔmsbB/ΔpagP strain and described the resulting OMVs as vesicles with reduced endotoxic activity [50]. This modification should be taken into account as part of the strategy adopted to mitigate the risks associated with LPS [116]. However, this does not eliminate the need for a more comprehensive assessment of immunological safety [98,117]. The desired immunostimulation is not, in fact, synonymous with a complete characterization of the immunogenicity and immunotoxicity of the carrier [98]. Further studies should make it possible to distinguish between a local anti-tumor response and undesirable systemic activation, and to assess the reproducibility of the composition and biological activity of subsequent batches of the preparation [98,118].

In systems that use living cells as carriers, the viability and function of the transport cell following the loading of the material are also key characteristics. Wen et al. demonstrated that neutrophils loaded with BNPD-Ce6 retained their viability and exhibited a migration capacity comparable to that of unloaded neutrophils in the Transwell assay [91]. These results provide direct evidence that the functions under investigation are preserved under the in vitro conditions used. Luo et al. utilized neutrophils isolated from bone marrow as living carriers for the bNe@AIE/Cas9-CD73 platform. The AIE dots showed good compatibility with the carrier cell, with neutrophil viability >80% at 100 μg/mL under non-irradiated conditions, whilst the inflammation-dependent transport property of the system was maintained in the in vitro BBB model. These results support the preservation of selected carrier functions, but do not constitute a complete functional characterization of neutrophils. The authors themselves highlighted the plasmid and nanomaterial loading efficiency, as well as the short in vitro lifespan of neutrophils, as limitations requiring further refinement [71]. From a translational perspective, the standardization of cell properties and phenotype, the amount of material loaded, and the functional stability of the prepared product over time remain important [119].

Platforms containing tools that directly interfere with the genome or protein levels, such as CRISPR/Cas9 and PROTAC, require further evaluation. In the bNe@AIE/Cas9-CD73 system, the CRISPR/Cas9 system was targeted at the NT5E gene, which encodes CD73, with the aim of reducing its expression and modifying the immunosuppressive tumor microenvironment. However, Luo et al. pointed out that potential off-target effects remain an issue requiring further investigation [71]. Further safety assessment should therefore distinguish between unintended editing outside the target genomic site and editing of the intended NT5E locus in non-target cells or tissues [120]. In the system described by Jiang et al., the however, spatio-temporal control of PROTAC activity was employed. The acidic endolysosomal environment caused the dissociation of PPT7 nanoparticles and the restoration of PPa’s photoactivity, whilst the singlet oxygen generated upon irradiation activated the prolec OT7 by cleaving a ^1O2-sensitive bond, leading to the release of ARV771 and the degradation of BRD4. The system was designed to limit the activation of the degradator both spatially and temporally; however, the data presented did not include a comprehensive assessment of degradation selectivity [21]. In the case of PROTAC, it is important to distinguish between the degradation of off-target proteins and the degradation of the target protein BRD4 in healthy cells or tissues, which corresponds to the potential on-target/off-tissue effect [121]. Further evaluation should therefore encompass both the selectivity of degradation at the proteome level and the distribution of the active degradator, as well as the degradation of the target protein in non-target tissues.

Some of the platforms analyzed combined PDT with solutions enabling the local delivery of therapy directly to the tumor. Jiang et al. used a surgically implanted, two-layer SOP MN microneedle system, designed for the local delivery of PPT7 nanoparticles containing the PROTAC prodrug and BSA-MnO_2_ nanoparticles [21]. Wu et al., on the other hand, developed a strategy utilizing a magnetic continuum robot (MCR), which was inserted into the cranial cavity via an access point created using the Ommaya system. This approach enabled the local delivery of a nanophotosensitizer and the delivery of light via an integrated optical fiber, whilst the robot’s positioning was controlled by an electromagnetic system and feedback mechanism [31]. These strategies introduce additional procedural and technological complexity; therefore, their translational assessment should cover not only the safety of the materials used and the method of light delivery, but also the risks associated with implantation or intracranial access, including tissue damage, bleeding and infection, as well as positioning accuracy, sterilizability and the reliability of the solution employed [122,123]. In the case of systems utilizing Ommaya access, the known complications associated with the implantation and use of the catheter take on added significance; these include, amongst other things, infection, bleeding, malposition or dysfunction. The further development of such technologies also requires defining their role in actual neurosurgical practice and assessing the feasibility and safety of the long-term or repeated local delivery of therapy [124].

The results to date, which indicate short-term tolerability, form the basis for further research, but do not replace an assessment tailored to the specific design and intended use. Priority should be given to determining the fate of the material and its metabolites, the preservation of the carrier’s biological functions, the selectivity of molecular action, and the safety of the route of administration. Distinguishing between established findings and unresolved risks allows for an assessment of translational readiness without equating promising therapeutic activity with proven clinical safety.

Based on the limitations identified across the reviewed studies, we propose a minimum preclinical evidence framework for assessing the translational readiness of multimodal PDT-based nanoplatforms for GBM (Table 2). This framework is not intended as a formal regulatory checklist or a rigid pass–fail system, but rather as a structured set of evidence domains that should be addressed before a platform is considered sufficiently characterized for further translational development. Importantly, the required depth of assessment should remain proportional to the composition, route of administration, mechanism of action, and intended clinical use of the individual platform.

## 6. Conclusions

The analysis of the 35 recent studies on multimodal PDT-based nanoplatforms for GBM demonstrates substantial progress has been made in the development of systems integrating targeted delivery, the modulation of the tumor microenvironment and complementary therapeutic mechanisms. However, the translational maturity of these platforms remains heterogeneous, and none of the multimodal PDT-based nanoplatforms covered in this review were identified as having progressed to clinical evaluation. This should be distinguished from PDT itself, which has already reached clinical investigation in GBM; rather, it indicates that the additional functionality introduced by multifunctional nanoplatforms has not yet been accompanied by a comparable level of translational validation. With regard to experimental models, 16 of the 19 in vivo studies used at least one orthotopic GBM model, indicating a clear shift towards more anatomically relevant settings. Nevertheless, clinically challenging scenarios, including post-resection disease, treatment-resistant tumors and recurrence after standard therapy, were represented only rarely, and direct comparisons with the current standard of care were generally lacking.

The ADME evidence was less mature than the efficacy data. Biodistribution was frequently assessed by fluorescence-based imaging, whereas quantitative pharmacokinetic parameters, tumor-to-healthy-brain comparisons, repeated-dose distribution studies and documented elimination pathways were reported only sporadically. In many cases, the available measurements characterized the behavior of selected platform components rather than the complete nanostructure, limiting conclusions regarding the in vivo fate and structural integrity of the complete system. Future studies should therefore combine complementary analytical approaches capable of distinguishing the distribution of individual labels or components from the behavior of the nanoplatform as a whole.

Safety evaluation was similarly dominated by short-term tolerability endpoints, including body weight, routine blood tests and terminal organ histology. Dedicated toxicology-oriented cohorts, serial laboratory measurements, neurological assessment, healthy-brain histopathology, repeated-dose evaluation and follow-up extending beyond the treatment period were much less common. Safety assessment should also encompass the phototherapeutic procedure itself, including appropriate light-only, nanoplatform-only and, where relevant, sham procedure controls, as well as the adequate characterization of irradiation conditions and potential thermal effects. Consequently, the absence of overt short-term toxicity should not be interpreted as evidence of established long-term systemic or neurological safety.

Platform-specific translational challenges further depend on material composition and delivery strategy. Inorganic systems require the characterization of persistence, transformation, chemical speciation and degradation products; polymeric and lipid-based carriers require the assessment of in vivo stability and premature cargo release; biomimetic and cell-derived systems require reproducible source-material characterization and the preservation of relevant biological functions; and locally implanted or device-assisted approaches require the evaluation of procedure-related risks, positioning reliability and compatibility with neurosurgical practice. For multifunctional systems, additional components should therefore be evaluated not only for their contribution to efficacy, but also for the new pharmacological, toxicological and manufacturing uncertainties that they introduce.

A clinically ready multimodal PDT nanoplatform for GBM would need to demonstrate substantially more than tumor growth inhibition. It should show durable therapeutic benefit in a clinically relevant orthotopic model, preferably under conditions reflecting the intended clinical setting; provide quantitative and mechanistically interpretable biodistribution, pharmacokinetic and elimination data; establish systemic, neurological and procedure-related safety using dedicated cohorts and sufficiently long follow-up; and demonstrate a clear therapeutic advantage over clinically relevant comparators. Equally importantly, the platform should have a well-characterized and reproducible composition, a feasible route of administration and light delivery, and a risk profile proportionate to its structural and functional complexity.

Overall, future progress should not depend on increasing platform complexity per se, but on ensuring that each additional functional component addresses a defined therapeutic, delivery or safety limitation, and is supported by appropriate experimental evidence. Priority should therefore be given to the rigorous characterization and comparative validation of the most promising systems. Once the core delivery, pharmacokinetic, safety and therapeutic properties of a platform have been established, further functional complexity—including stimulus-responsive or light-controlled mechanisms that improve spatial or temporal selectivity—may be justified when it provides a measurable therapeutic or safety advantage without introducing disproportionate translational barriers. In this context, increasing complexity and translational validation should be viewed as sequential and complementary steps, rather than opposing development strategies.

## Figures and Tables

**Table 1:** Preclinical validation profile of the 19 multimodal PDT-based nanoplatforms evaluated in vivo for glioblastoma.

Ref.	Nanoplatform	Tumor In Vivo Model and Host	Administration Route	Survival	Biodistribution/PK/Clearance Evidence	Main Translational Consideration
[22]	MLCNPs	Orthotopic GL261-Luc; additional subcutaneous model used for imaging; immunocompetent	i.v.	No	Serial fluorescence imaging and ex vivo organ imaging; quantitative PK and clearance NR	Immunocompetent orthotopic model, but limited pharmacokinetic characterization
[70]	MET/Ce6@Lipo@CM@T10	Orthotopic U87-Luc; immunodeficient	i.v.	No	Serial fluorescence imaging and ex vivo organ imaging; PK and clearance NR	Intracranial evaluation of biomimetic targeting, but small experimental groups and no survival analysis
[75]	Pt@PCN-222(Mn)-PEG	Orthotopic U87-Luc; immunodeficient	i.v.	Yes	Fluorescence imaging, ICP-MS analysis of Mn, blood PK analysis, and Mn detection in urine and feces	One of the most extensive ADME datasets; elemental measurements do not establish clearance of the intact nanoplatform
[56]	F8BT-PtOEP CPNs/F8BT-PtOEP-Tf CPNs	Orthotopic U87MG-tdiRFP; immunodeficient	i.v.	Yes	Quantitative ICP-MS analysis of Pt in brain and liver at 24 h; PK and clearance NR	Quantitative component-level tissue analysis, but Pt detection does not demonstrate intact-particle localization
[57]	HD-P@BM	Orthotopic GL261; immunocompetent	i.v.	Yes	Fluorescence-based brain/tumor distribution; quantitative PK and clearance NR	Combines an immunocompetent orthotopic model with relatively extensive short-term biosafety evaluation
[71]	bNe@AIE/Cas9-CD73	Orthotopic GL261-Luc; immunocompetent	i.v.	Yes	NIR-II imaging, ex vivo organ distribution and quantitative blood half-life	Relatively comprehensive in vivo validation; long-term and gene-editing off-target safety remain unresolved
[31]	MCR + PNPs	Orthotopic Sleeping Beauty-derived GBM; immunocompetent	Local intracranial delivery	No	Regional brain distribution assessed; classical systemic PK and clearance NR	Bypasses systemic BBB/BBTB delivery but introduces device- and procedure-specific translational challenges
[32]	Mag-Diatoms	Orthotopic Sleeping Beauty-derived mGSC model; immunocompetent	Local intracranial administration	No	Systemic quantitative biodistribution, PK and clearance not established	Local magnetic delivery limits extrapolation to systemic pharmacokinetics and distribution
[21]	SOP MN: PPT7 + BSA-MnO_2_	U87-MG subcutaneous and orthotopic models; immunodeficient	Local microneedle implantation	Yes	Classical systemic PK and clearance NR	Local multimodal delivery was tested, but the orthotopic experiment was not a post-resection model
[91]	BNPD-Ce6@NE	Orthotopic GL261-Luc; immunocompetent	i.v.	Yes	Ce6-based fluorescence imaging and ex vivo organ assessment; PK and clearance NR	Cell-mediated delivery evaluated in an intact immune system, but quantitative ADME characterization was limited
[18]	Cela@Lip-IRH	Subcutaneous C6 xenograft; immunodeficient	i.v.	No	Serial fluorescence-based distribution; quantitative PK and clearance NR	Proof-of-concept efficacy without evaluation of intracranial delivery or BBB/BBTB constraints
[46]	ApoE-P3 NPs	Orthotopic U251-TR; TMZ-resistant model; immunodeficient	i.v.	Yes	NIR-II distribution and quantitative blood half-life	Incorporates experimentally confirmed TMZ resistance, but pre-existing cellular resistance is not equivalent to recurrent GBM after prior therapy
[50]	FBFO@HM@aOPN ± anti-PD-1	Orthotopic CT2A and post-resection CT2A; immunocompetent	i.v.	Yes	Cy5.5-based fluorescence biodistribution; quantitative classical PK and clearance NR	Only clearly identified post-resection model in the reviewed set; particularly relevant to immunomodulatory therapy
[19]	Fe_3_O_4_@PDA@Ru	Subcutaneous U87-MG; immunodeficient	i.v.	No	Quantitative biodistribution, PK and clearance NR	Multimodal efficacy demonstrated only in a subcutaneous model
[63]	OT@COF-RVG	Orthotopic GL261-Luc; immunocompetent	Therapeutic route not clearly specified	Yes	Quantitative PK and clearance NR; biodistribution of the final therapeutic formulation not clearly established	Immunocompetent intracranial model, but the administration route of the main therapeutic cohort remains insufficiently specified
[94]	Empa@IRT	Orthotopic LN229/LN229-Luc; immunodeficient	i.v.	Yes	Serial fluorescence imaging; quantitative PK and clearance NR	Includes neurological and behavioral assessment, but quantitative pharmacokinetic characterization is lacking
[100]	Er-LnNPs@QDs	Orthotopic U87MG-Luc; immunodeficient	i.v.	Yes	NIR-II imaging and ICP-MS analysis of Ag and Y^3+^ in brain tissue; PK and clearance NR	Quantifies selected components, but does not establish the disposition of the intact nanoplatform
[52]	MeTTh NPs-Tf + FUS	Small orthotopic GFP-GL261 tumor; immunocompetent	i.v. + FUS	No	Local two-photon brain imaging; no complete systemic biodistribution or quantitative PK	Evaluation in a small intracranial tumor is valuable, but cranial-window/FUS procedures affect translational interpretation
[20]	CD@EXO_msc_	Subcutaneous U87MG; immunodeficient	i.v.	No	IVIS fluorescence at 24 h; quantitative PK and clearance NR	Exosome-associated delivery demonstrated in vivo, but therapeutic efficacy was not evaluated in an intracranial GBM model

Abbreviations: BBB, blood–brain barrier; BBTB, blood–tumor barrier; FUS, focused ultrasound; i.v., intravenous; ICP-MS, inductively coupled plasma mass spectrometry; NR, not reported; PK, pharmacokinetics; TMZ, temozolomide.

**Table 2:** Proposed minimum preclinical evidence framework for assessing the translational readiness of multimodal PDT-based nanoplatforms for glioblastoma.

Domain	Minimum Evidence Recommended Before Translational Advancement	Key Question
Model relevance	Therapeutic efficacy demonstrated in an orthotopic GBM model; use of an immunocompetent model when immune mechanisms contribute to efficacy; inclusion of post-resection, treatment-resistant or recurrent-disease settings when clinically relevant to the intended use	Does the model reproduce the anatomical, biological and therapeutic context in which the platform is intended to be used?
Therapeutic benchmarking	Appropriate untreated/vehicle and light controls, component controls where needed, and comparison with clinically relevant standard-of-care therapy or a justified reference treatment	Does the nanoplatform provide benefit beyond its individual components and current therapeutic benchmarks?
Biodistribution and PK	Serial blood/plasma measurements for systemically administered platforms; quantitative tumor, healthy brain and major-organ distribution; appropriate time-course analysis; assessment after repeated dosing when applicable	Where does the platform go, for how long, and does repeated administration result in accumulation?
Nanoplatform integrity and fate	Analytical strategy capable of distinguishing an intact platform from a released tracer, payload, ion or degradation product; preferably complementary tracking of carrier and cargo	Does the detected signal represent the complete nanoplatform or only one of its components?
Elimination and degradation	Characterization of major elimination pathways and degradation/transformation products, including prolonged follow-up when persistent materials are used	Can the material and its biologically relevant transformation products be cleared or otherwise accounted for?
Systemic safety	Dedicated safety cohort with serial hematology and clinical chemistry, major-organ histopathology, repeated-dose assessment where applicable, and follow-up extending beyond the treatment period; approximately 4 weeks of post-treatment observation is proposed as a pragmatic minimum, with longer follow-up for persistent materials	Is systemic tolerability demonstrated independently of antitumor efficacy?
Neurological safety	Assessment of healthy brain tissue and neurological/behavioral function, including light-only, nanoplatform-only and, where applicable, sham procedure controls, to distinguish treatment toxicity from tumor-related neurological deterioration and procedure-related injury.	Does treatment damage normal brain tissue or neurological function?
Immune safety	Evaluation appropriate to platform composition and mechanism, particularly for biomimetic, cell-derived, EV-based and immunomodulatory systems	Does repeated exposure cause inflammatory, immunogenic or other immune-mediated adverse effects?
Durability of response	Survival analysis or another justified long-term disease-control endpoint, with follow-up extending beyond the immediate treatment period	Is tumor suppression durable rather than only transient?
Reproducibility and reporting	Clearly reported sample sizes, dosing schedule, administration route, irradiation parameters, exclusions, randomization/blinding where applicable, and predefined endpoints	Can the experiment and its interpretation be independently reproduced?

Abbreviations: EV, extracellular vesicle; GBM, glioblastoma; PDT, photodynamic therapy; PK, pharmacokinetics.

## Data Availability

No original experimental data or new datasets were generated in this review. All data and information discussed were derived from previously published studies cited in the manuscript.
